# Individual differences provide a nuanced understanding of the contributions of age, experience, and level played to superior perceptual-cognitive-motor skill

**DOI:** 10.3389/fpsyg.2025.1470789

**Published:** 2025-04-22

**Authors:** Khaya Morris-Binelli, Minerva A. Westbrook, Benjamin Piggott, Sean Müller, Paola Chivers

**Affiliations:** ^1^School of Health Sciences, The University of Notre Dame Australia, Fremantle, WA, Australia; ^2^Centre for Smart Analytics, Federation University, Ballarat, VIC, Australia; ^3^Institute for Health Research, The University of Notre Dame Australia, Fremantle, WA, Australia; ^4^School of Medical and Health Sciences, Edith Cowan University, Joondalup, WA, Australia

**Keywords:** expertise, field test, Rugby union, invasion sport, decision-making

## Abstract

Theories of expertise either predict superior performance is due to monotonic and progressive exposure to a domain task or due to non-linear exposure to a domain. The aim of this study was to explore the predictions of these theories by use of an individual differences approach to investigate how age, experience, and level played within a sample of athletes with high expertise contributes to superior perceptual-cognitive-motor skill. Twenty-seven players sampled from junior rugby union high-performance pathways and professional rugby union teams in Australia completed an in-situ perceptual-cognitive-motor test involving four attackers and three defenders. Participants were presented with scenarios representative of a typical game and had to decide whether, and who, to pass the ball, execute the pass, or run with the ball. Performance was scored based upon an expert coach rating scale. Results indicated significant individual differences were more pronounced for decision-making, than for motor execution components of the task. Superior decision-making was not dependent solely upon greater experience in playing rugby union, nor age or level played. Further, superior decision-making was not solely dependent upon those participants who specialized in positional play during the typical game scenarios. Findings indicate that theories of expertise may need to accommodate that prolonged exposure to a domain does not provide a complete explanation of expert performance and that the capability to make effective decisions is highly individualized.

## 1 Introduction

Perceptual-cognitive-motor skills are regarded as vital for athletes to achieve exceptional performance (Morris-Binelli et al., 2020). The perceptual-cognitive component involves use of information to predict and decide upon action, while the motor component involves execution of what has been decided (Morris-Binelli et al., 2020). Two important perceptual-cognitive-motor skills that discriminate expert sport performance are visual anticipation and decision-making (Williams and Jackson, 2019). In team sports such as rugby union, soccer, and Australian rules football, these skills are crucial because performers are faced with multiple teammate and opponent positional changes over short periods of time. Therefore, performers in possession of the ball must quickly detect changes to opposition player and teammate positioning for accurate anticipation and decision-making in order to maintain control, or dispose of, the ball efficiently (Sherwood et al., 2019; Ashford et al., 2021). For example, in rugby union, when a team is in an attacking phase of the game, forward attacking players attempt to advance the ball into the oppositions part of the field by retaining possession of the ball and pushing against opposition defending players to make ground and score points. In contrast, back attacking players pass the ball more frequently to their teammates and move the ball in a lateral and forward direction to create space and run in-between the opposition to score (Campbell et al., 2018). Both forward and back attacking players are faced with opposition players that attempt to tackle them directly from in front or the side in order to dispose them of the ball. Therefore, in rugby union, a player in possession of the ball needs to swiftly read where opposition players are positioned, predict where they may move to next, decide whether to run with the ball, make contact with an opponent(s), or pass the ball to a teammate to gain ground in order to position their team to score (Campbell et al., 2018).

Visual anticipation is the capability of a performer (e.g., rugby union player) to utilize contextual (e.g., opponent action tendencies, positioning of other players on the field) and direct opponent kinematic information (i.e., movement patterns) to predict possible outcomes and guide an efficient motor response (Navia et al., 2013; Morris-Binelli and Müller, 2017; Gredin et al., 2018; Williams and Jackson, 2019; Morris-Binelli et al., 2021a). Decision-making involves a choice made such as to pass, dribble, or maintain possession of the ball in rugby, soccer, or basketball, respectively, based upon the pick-up of information during the anticipatory phase, to respond effectively (Gabbett et al., 2008; Ashford et al., 2021). To understand anticipation and decision-making, research has frequently used an Expert Performance Approach (Ericsson et al., 2007) where comparisons are made between a group of performers with higher expertise and a group of performers with lower expertise (usually novices) on domain-specific tasks (e.g., Lorains et al., 2013; Ashford et al., 2021). Such studies employing a video simulation paradigm have reported athletes with higher expertise were superior to athletes with lower expertise in the recall, recognition, and prediction of structured, but not unstructured, patterns of play to make more accurate decisions (Allard et al., 1980; Helsen and Starkes, 1999; Gorman et al., 2011). This superior capability is due to prior exposure to structured patterns of play within domain or non-domain sports that is stored and retrieved from long-term memory for skill execution (Baker et al., 2003). Therefore, by anticipating future positioning of teammates and opponents, athletes with higher expertise create time to be able to make fast and accurate decisions (Gorman et al., 2013; Sherwood et al., 2019; Ashford et al., 2021). Due to the importance of anticipation and decision-making for superior performance in sport, a key theoretical consideration in the literature has been understanding how domain specific expertise and experience contributes to the development of these skills (Sherwood et al., 2019).

Ericsson et al. (1993) deliberate practice theory has been predominantly relied upon to explain expert performance. It predicts that progressively increasing monotonic exposure to a domain (i.e., experience in terms of years of participation) contributes to the development of expert domain-specific skill such as perceptual-cognitive-motor skill. Expert performance can be defined as attainment of an exceptional level of participation, such as national and international level competition (Baker et al., 2015). Studies that have investigated this topic in sport have reported that athletes with higher expertise have more elaborate declarative knowledge for superior decision-making, compared to athletes with lower expertise, which can be developed through domain (Williams and Davids, 1995) and non-domain (Baker et al., 2003; Güllich et al., 2022) specific experiences. Therefore, athletes with higher expertise and more experience, as well as those who specialize in specific positions on a team such as the backs in rugby union (see Campbell et al., 2018), are proposed to be better able to utilize contextual and kinematic information to facilitate superior decision-making, than athletes with lower expertise and less experience. An alternative perspective of expertise to Ericsson et al. (1993) deliberate practice theory proposes that the acquisition of perceptual-cognitive-motor skills is non-linear in nature (Chow et al., 2016; Pacheco and Newell, 2018). This suggests that progressive increase in domain-specific experience does not necessarily lead to superior perceptual-cognitive-motor skill, but rather it is the capability, independent of experience, to better use dynamically evolving information sources to guide decision-making. Accordingly, use of the Expert Performance Approach where comparisons are typically made between extremes of the skill continuum to understand superior decision-making, may not have the sensitivity to detect subtle differences in performance capability and evaluate these theoretical predictions (Glazier, 2017).

A limitation of the Expert Performance Approach paradigm, where performers with higher expertise are compared to performers with considerably lower expertise, is that these groups can differ considerably in both participation level attained and experience in the domain task. For example, in some pattern recall and anticipation studies (e.g., Helsen and Starkes, 1999; Müller et al., 2010; Gorman et al., 2011), the expert group had played at a higher level (e.g., regional, national, or international) and had greater experience (e.g., 10 years) in the sport, compared to the group of performers with lower expertise (e.g., amateur players, university students). Accordingly, it is difficult to determine whether, and to what degree, experience and/or playing level influence expert perceptual-cognitive-motor skill within samples of higher expertise. Further, such a paradigm limits the capability to understand the influence that age has on expert performance. An early study that investigated the contribution of maturation to anticipation used an age-matched design and reported that expert superiority is evident only at the adult age (Abernethy, 1988). Again, however, at each age group, experts with significantly greater experience were compared to novices with considerably less experience in the sport (Abernethy, 1988). More recent studies have attempted to compare different age groups of players within a developmental pathway, and therefore at closer stages of the skill continuum, on anticipation and decision-making (De Waelle et al., 2021; Murr et al., 2021). De Waelle et al. (2021) indicated that superior anticipation or decision-making was apparent at under 17 or adult age groups. Murr et al. (2021) used a video-based task to investigate the decision-making capability of under 16, under 17, and under 19 soccer players within a high-performance development pathway. In line with the predominant perspective of expertise (Ericsson et al., 1993), under 17 and under 19 players significantly outperformed under 16 players. However, there were no significant differences in decision-making between under 19 and under 17 players. A potential reason for this could be due to the limited sensitivity of group-based comparisons to detect differences in decision-making skill when investigating athletes at closer stages of the skill continuum (Murr et al., 2021). A further reason could be that superior decision-making is due to non-linear learning across age (Chow et al., 2016; Glazier, 2017). Therefore, an alternative paradigm than group-based investigations may provide further understanding on the contribution of level of attained play (expertise), number of years of competitive play (experience), and age on perceptual-cognitive-motor skill (Glazier, 2017; Murr et al., 2021).

One way to overcome the limitation of the Expert Performance Approach group paradigm is to use an inter-individual differences approach, where direct comparisons can be made between the age/experience/level played profile of each athlete where both level of play attained and experience do not vary at extremes (Glazier, 2017; Nickels et al., 2022). Early expertise studies into pattern recall and decision-making indeed used case study or inter-individual difference comparisons (e.g., Chase and Simon, 1973). These studies reported that individual performance of grandmaster chess players were superior to players with lower expertise (e.g., Class A) in their capability to recall structured plays for superior decision-making (Chase and Simon, 1973). Sport researchers have used individual differences designs to understand the underpinning mechanism of expertise. Such studies have reported that some national level table tennis players made fine adjustments to their bat to intercept a ball (Bootsma and van Wieringen, 1990), and a national level cricket batter, but not a club level player, made anticipatory saccades to strike a fast ball (Land and McLeod, 2000). More recently, Morris-Binelli et al. (2021a) reported that some, but not all, national level field hockey goalkeepers could more accurately anticipate the goal location of a drag-flick than some, but not all, international level goalkeepers. These results suggest that superior perceptual-cognitive skill is not solely depended upon progressively higher level played. Moreover, these studies have predominantly focused upon non-sport tasks or striking sports, with a lack of focus upon invasion sports such as rugby union.

Investigating perceptual-cognitive-motor skill using an inter-individual differences paradigm is important from theoretical and practical perspectives. In relation to theory, an inter-individual differences paradigm incorporating athletes with high expertise and experience may provide a more nuanced understanding of the extent to which experience, age, or level played contribute to this vital skill (Glazier, 2017). From a practical perspective, a better understanding of the nature of perceptual-cognitive-motor skill could inform talent identification, as well as coaches’ tactical decisions. For example, in rugby union, players are typically characterized as either *backs* or *forwards*, with the backs predominately responsible for making decisions regarding which teammate to pass the ball to and then executing the pass to score (see Campbell et al., 2018). If, however, some forwards have similar perceptual-cognitive-motor capabilities to backs, despite less position-specific experience, then coaches could have a tactical advantage by having more players to call upon to increase the team’s chances of success. The purpose of this study was to investigate how age, experience, and level played contributes to perceptual-cognitive-motor skill using a fine-grained inter-individual differences paradigm. Rugby union players who were members of a high-performance development and professional program participated in field-based simulated scenarios of four attackers versus three defenders typical of a match. Their decision-making and motor skill execution were assessed through criteria established by expert rugby union coaches. Based upon the literature discussed above, we predicted that increased age, as well as greater experience, particularly in the position specific decision-making scenario, and attained expert level in rugby union would contribute to superior decision-making.

## 2 Materials and methods

### 2.1 Participants

Twenty seven rugby union players (*n* = 25 male, *n* = 2 female) aged between 14 and 29 years (*M*_age_ = 20, *SD* = 3.54) were recruited from a state academy team (SA; *n* = 10), a professional academy team (PA; *n* = 11), a professional senior team (PS; *n* = 4), and an international team (INT; *n* = 2) in Australia. According to Baker et al. (2015) taxonomy for skill in sport, these participants can be considered advanced (academy) and expert (professional senior and international) players. Based upon expert coach advice and player self-report, participants were further classified into playing category, position, and number that is common to positional play on a rugby union team (see Table 1). This was done to be able to identify those players who were specifically considered as *playmakers* in decision-making positions. For example, the playmakers on a rugby union team are typically classified as the backs (inside and outside back), such as number 9 and 10 who are required to make the critical decision to pass the ball from the ruck (Campbell et al., 2018; Pastor et al., 2023). Therefore, it was possible to identify players within the sample who not only had greater experience in rugby union, but also those who specialized in decision-making specific positions of play. Demographic details of all participants including age, experience, and highest participation level attained are presented in Table 1. As our design was a nested inter-individual differences comparison, an a-priori power analysis conducted in G*Power (Version 3.1.9.7) with *α* = 0.05, 80% power, and 95% confidence interval, for a repeated measures analysis, indicated that 27 participants with 32 trials each (864 trials in total) could detect a small effect of *f* = 0.12 (Morris-Binelli et al., 2021a). Ethical approval was obtained through the participating institutional ethics committees (Reference Number: 2021-136F). Participants provided written informed consent, and for those participants under the age of 18, a parent or guardian also provided written informed consent.

**Table 1:** Demographic details of participants including age, expertise level, playing level (team), experience, highest level attained, and position of play.

Player ID	Age	Expertise level	Playing level (Team)	Experience (Years playing competitive rugby)	Highest participation level	Playing position (Playing number)	Playing category	Years in playing position
1	14	Advanced	SA	7	State	Tighthead Prop (3)	Tight Forward	5
2	18	Advanced	SA	13	State	Scrum-half (9)	Inside Back	7
3	17	Advanced	SA	11	State	Lock (4, 5)	Tight Forward	6
4	15	Advanced	SA	8	Regional	Inside Center (12)	Inside Back	1
5	16	Advanced	SA	8	State	Blindside Flanker (6)	Loose Forward	8
6	14	Advanced	SA	7	State	Scrum-half (9)	Inside Back	2
7	18	Advanced	SA	9	State	Left Wing (11)	Outside Back	9
8	18	Advanced	SA	13	Club	Scrum-half (9)	Inside Back	4
9	16	Advanced	SA	10	Regional	Inside Center (12)	Inside Back	3
10	19	Advanced	PA	2	Club	Full Back (15)	Outside back	2
11	21	Advanced	PA	10	State	Blindside Flanker (6)	Loose Forward	5
12	19	Advanced	PA	12	National	Number 8 (8)	Loose Forward	8
13	22	Advanced	PA	16	State	Fly-half (10)	Inside Back	6
14	23	Expert	PS	10	Professional	Outside Centre (13)	Inside Back	3
15	20	Advanced	PA	2	State	Number 8 (8)	Loose Forward	2
16	20	Advanced	PA	17	Professional	Loosehead Prop (1)	Tight Forward	4
17	21	Expert	PS	12	Professional	Left Wing (11)	Outside Back	3
18	20	Advanced	PA	14	Club	Loosehead Prop (1)	Tight Forward	4
19	21	Advanced	PA	9	National	Left Wing (11)	Outside back	9
20	27	Expert	PS	15	International	Hooker (2)	Tight Forward	15
21	19	Advanced	PA	9	State	Blindside Flanker (6)	Loose forward	6
22	21	Advanced	PA	12	Club	Tighthead Prop (3)	Tight Forward	6
23	20	Advanced	PA	16	Professional	Number 8 (8)	Loose Forward	7
24	18	Advanced	SA	8	State	Full Back (15)	Outside back	6
25	29	Expert	INT	11	National	Fly-half (10)	Inside Back	5
26	19	Expert	INT	5	National	Openside Flanker (7)	Loose Forward	3
27	26	Expert	PS	22	Professional	Scrum-half (9)	Inside Back	16

Expertise levels classified as per Baker et al. (2015). SA, State Academy; PA, Professional Academy; PS, Professional Senior; INT, International.

### 2.2 Field test design

**Figure 1:**
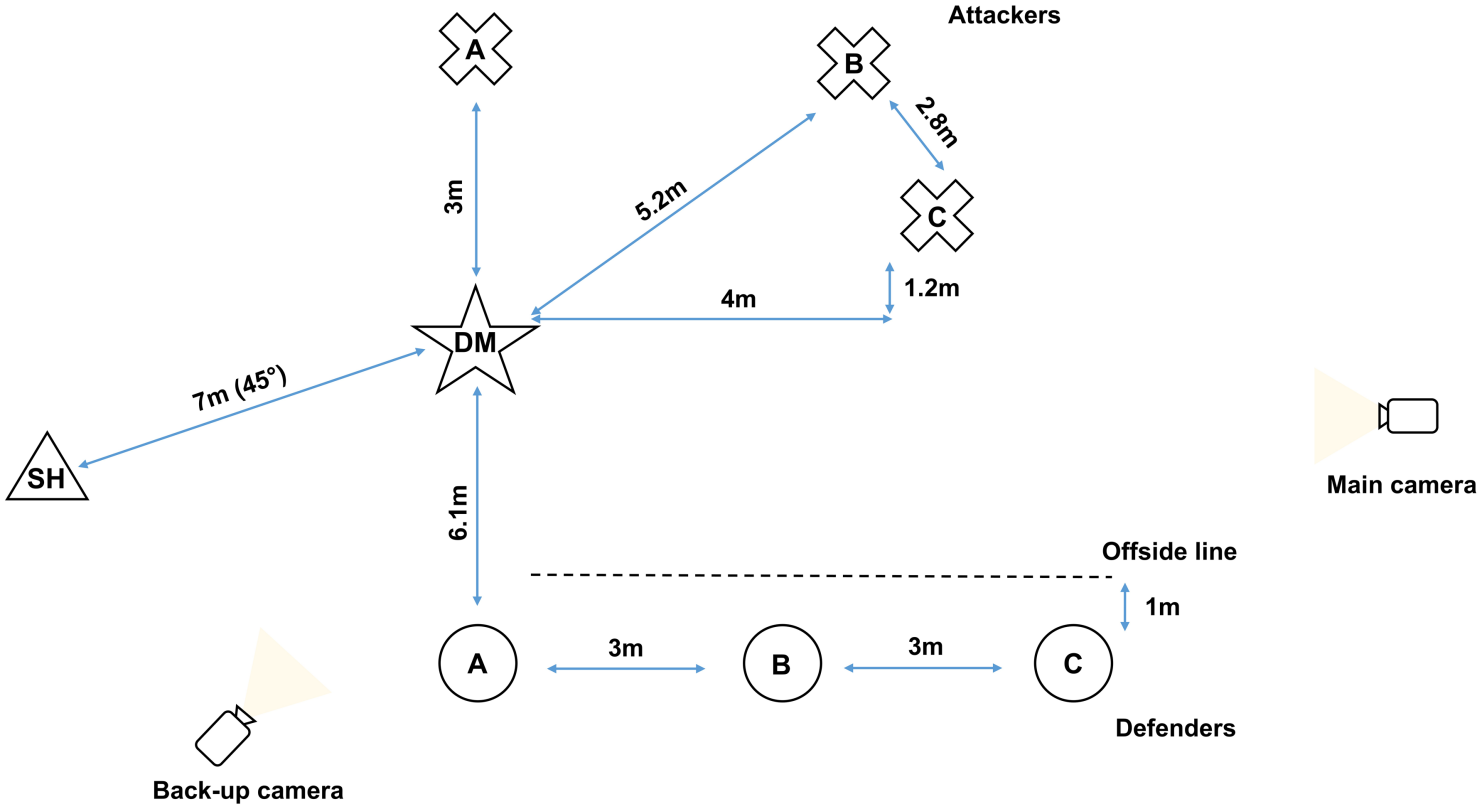
Rugby union decision-making test setup for passing to the left. A, B, and C within the crosses are attacking players. A, B, and C within the circles are defending players. SH refers to the scrum-half and DM refers to the decision-maker who is also an attacking player.

The field test design utilized the concept of a representative task that included in-situ perceptual information and full body movement to assess the decision-making and execution capability of the participants (PAC-6; Huesmann and Loffing, 2024). A representative task includes perceptual and/or motor components of skill that relate to the context of generalization, which in this paper is an aspect of competition (Araújo and Davids, 2015). Two synchronized GoPro (HERO6 Black) cameras sampling at 120 frames-per-second were used to capture all trials. The main camera was positioned on the decision-maker’s (DM) passing side of the play area. The back-up camera was positioned behind the defenders at 45° on the DM’s non-passing side. Both cameras were fastened to tripods at heights of 126 cm. Coding during analysis mainly relied on footage captured by the main camera, while the back-up camera provided an alternative viewpoint of the trials should the view in the main camera be obscured (see Figure 1).

Each trial involved the DM with seven other players. This consisted of the DM and three teammates who attack three defensive players. The final player (scrum-half) *fed* the ball to the DM from the base of the ruck, consequently, signaling the start of each trial (see Figure 1). These players were also of an advanced and expert skill level as per Baker et al. (2015). For each trial, unbeknown to the DM and the scrum-half, the three attacking and three defending players ran one of four play variations (see Supplementary Figures 1–4). These play variations were developed in consultation with professional expert coaches from both the state academy and professional academy. These coaches had an average of 6.5 years (*SD* = 0.5 years) of professional rugby union coaching experience at both state and national levels in Australia. Play development consisted of the coaches discussing common running patterns that defending and attacking players perform in *off the ruck* match play scenarios until they agreed upon four play variations that they believed accurately captured common player movements. Off the ruck scenarios usually evolve from a tackle and they are a strategy used by attackers to engage defenders thereby creating space and opportunity to advance down field toward the try lines (i.e., behind the defenders). Once the attacker (the DM) receives the ball from the scrum-half, they are required to decide the next move based on the actions of their teammates (attackers) and the opposition defenders. For each variation, the DM could: (i) pass to attacker A, (ii) pass to attacker B, (iii) pass to attacker C, or (iv) run through the defensive line without passing.

Based upon consultation with two professional expert coaches (Piggott et al., 2019; Murr et al., 2021), each play variation was scored such that two of the options were categorized into *good* decisions and two options were categorized into *poor* decisions. For each play, the coaches together evaluated the effectiveness of each option to progress the ball toward the try line to score. Any differences in the evaluation of options were further discussed until 100% agreement was reached (Piggott et al., 2019; Murr et al., 2021). A good decision was awarded a score of one and a poor decision was awarded a score of zero (see Supplementary Table 1). In addition to the decision-making score, the DM’s skill execution was also scored on each trial. For this component, two sets of criteria were devised within the rating of skill execution, specifically, a passing criterion and running criterion (in the latter, the athlete decided to run forward instead of passing the ball to a teammate). The two expert coaches discussed the key elements of a good and poor execution of a pass and run until 100% agreement was reached (Piggott et al., 2019). A *good* execution was awarded a score of one, while a *poor* execution was awarded a score of zero (see Supplementary Table 1). For each trial, participants were also assigned a *total* score, which was a combination of decision and execution scores. To receive a total score of one for each trial, which represented a *good* total score, participants had to receive a score of one for both decision and execution. This was because the total score was defined as a good decision and execution in the same trial and is representative of optimal performance during a game (Piggott et al., 2019).

To ensure that the test was highly representative to the match setting, each participant completed trials where they had to pass the ball to the left and right side of their body. Accordingly, each participant completed a total of 32 trials, consisting of 4 play variations × 2 passing sides × 4 attempts, with 16 trials on each passing side. To minimize familiarization with the test stimuli, the order of defensive variations was randomized for each participant. Further, the order that each participant completed passes to the left or right side was counterbalanced to reduce order effects.

### 2.3 Testing procedure

Prior to the field test, each participant completed a demographic questionnaire that asked for their age, highest level of participation, years of participation in competition, playing position, and years of competitive experience in that position. During each testing session, which consisted of two participants (i.e., DM) individually completing the test, the area of play was set up based upon pre-determined measurements (see Figure 1) and the cameras were positioned and synchronized. Each testing session consisted of two participants completing the field test due to the limited availability to convene the required number of players around their other training commitments (Müller et al., 2015). Before the test proper, the DM was informed that on each trial, they would be required to either pass the ball to the most suitable teammate (attacker) or run forward with the ball and that their teammates (attackers) and defenders would be running several different variations. Prior to each trial, the attackers and defenders met with the experimenter (positioned next to the main camera, see Figure 1) to be informed of the play variation to run. These players were also informed to run each play with match-like intensity.

When 16 trials were completed on the pre-determined passing side, the athletes had a short reprieve as the cameras were re-positioned to optimally capture trials on the other passing side. If a trial was not completed successfully (e.g., the ball was not successfully passed from the scrum-half to the DM), the next trial in the test matrix was completed and the unsuccessful trial was repeated later. The test took each participant approximately 15 min to complete. Later, video footage was reviewed by the second author (MAW), and each trial was awarded a score of zero or one for decision, execution, and total accuracy. The reliability of scoring was assessed by inter-rater reliability, whereby the first author (KM-B) independently scored 192 (22%) randomly selected trials using the video record.

### 2.4 Dependent measures and statistical analyses

To address the hypothesis, the data was analyzed using two approaches in line with current literature (Chomienne et al., 2024). First, the overall influence of the independent variables age, experience, and playing level at a group level on the dependent variables good/poor (a) decision-making accuracy, (b) execution accuracy, and (c) total accuracy were investigated. Second, an inter-individual differences analysis was used to further probe differences in good/poor decision-making accuracy, execution accuracy, and total accuracy, relative to each individual athlete’s own age, experience, and playing level profile. As this main aspect of the study investigated inter-individual differences in performance, the independent variable was each participant (i.e., each DM). Data are plotted as percentages for ease of graphical display.

Statistical analysis was performed using IBM SPSS Statistics (Version 27). Generalized Estimating Equations (GEE) using a binomial probability distribution with Logit link function were run to investigate the hypothesis. GEE’s were used as they allow for the correct modeling of repeated observations and allow for non-normal distribution models (Ghisletta and Spini, 2004). These models do not require normality of residuals (Ballinger, 2004). For the overall group-level analyses, age, experience, and playing level were included in the same model as fixed effects, while trials (i.e., 32 per participant) were included as a repeated factor to account for the repeated trials completed by each participant. Tests of model effects (Wald χ^2^ and *p*-value) were reported for each fixed effect. Parameter estimates were reported as odds ratios (OR) with 95% Wald confidence intervals. For the inter-individual differences analyses, individual participants were included as a fixed effect and trials were included as a repeated factor. All GEE analyses were run with alpha levels set at 0.05. Bonferroni adjusted post-hoc pairwise comparisons examined between player differences in order to lower the significance threshold and the likelihood of Type I errors (McLaughlin and Sainani, 2014). Wald 95% confidence interval was calculated for mean differences between participants.

The reliability of scoring the field test was assessed using inter-rater reliability which was calculated using Cohen’s kappa (ĸ; Cohen, 1960; Denham, 2017). As per Cohen (Cohen, 1960), the following benchmarks of agreement between scorers were used for kappa coefficient values: 0.01–0.20 (none to slight), 0.21–0.40 (fair), 0.41–0.60 (moderate), 0.61–0.80 (good), and 0.81–1.00 (excellent). Results of these analyses for decision-making (ĸ = 0.91, *p* < 0.001) and execution (ĸ = 0.89, *p* < 0.001) scores indicated excellent levels of agreement. Inter-rater reliability also demonstrated an excellent level of agreement for total scores, ĸ = 0.93, *p* < 0.001.

## 3 Results

### 3.1 Decision-making accuracy

#### 3.1.1 Overall influence of age, experience, and playing level

**Figure 2:**
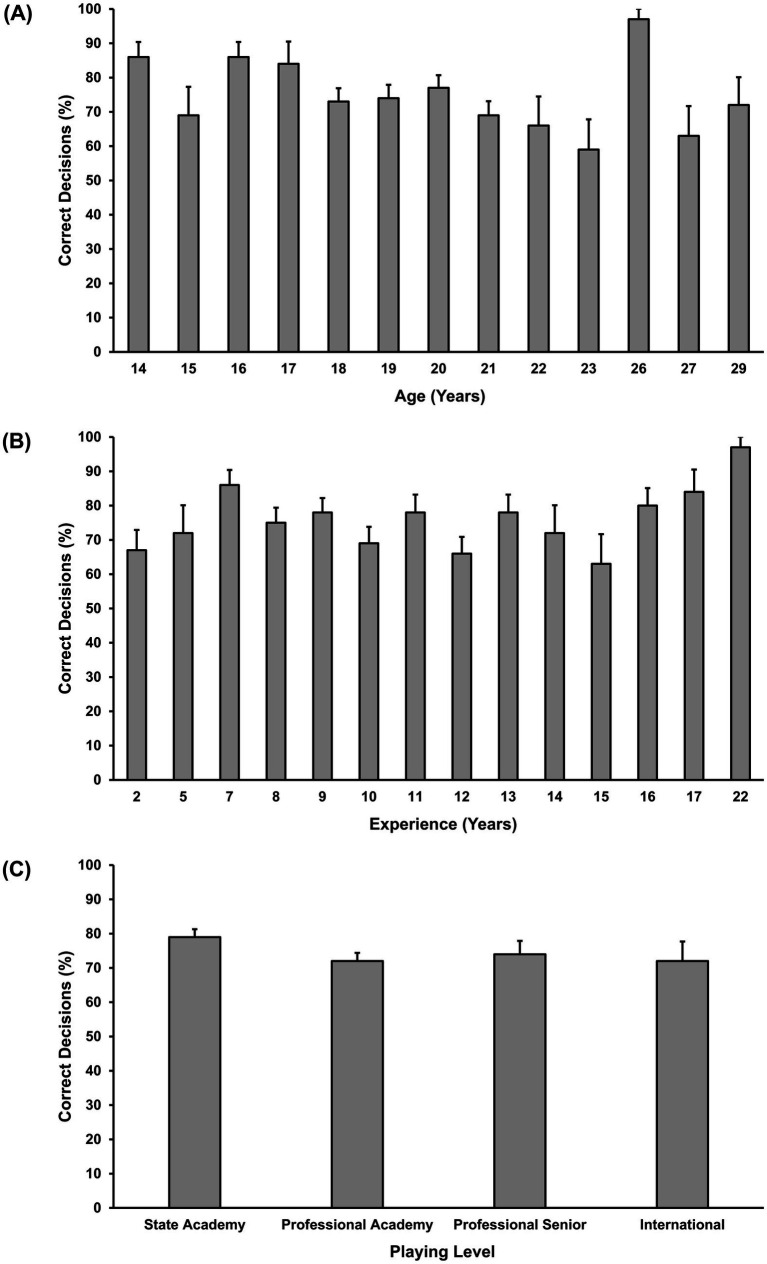
Mean percentage correct for decision-making relative to age **(A)**, experience **(B)**, and playing level **(C)**. Error bars indicate standard error.

Figure 2 presents mean decision-making scores relative to age, experience, and playing level. GEE indicated a significant effect of age [χ^2^ (1) = 4.75, *p* = 0.029] and experience [χ^2^ (1) = 13.88, *p* < 0.001], but not playing level [χ^2^ (3) = 3.28, *p* = 0.350] on decision-making. An increase in age by 1 year showed an 11% decrease in the odds of making an accurate (good) decision (OR = 0.895, 95% CI 19–1%), while an increase in experience by 1 year showed an 8% increase in the odds of making an accurate (good) decision (OR = 1.08, 95% CI 4–12%).

#### 3.1.2 Inter-individual differences comparison

**Figure 3:**
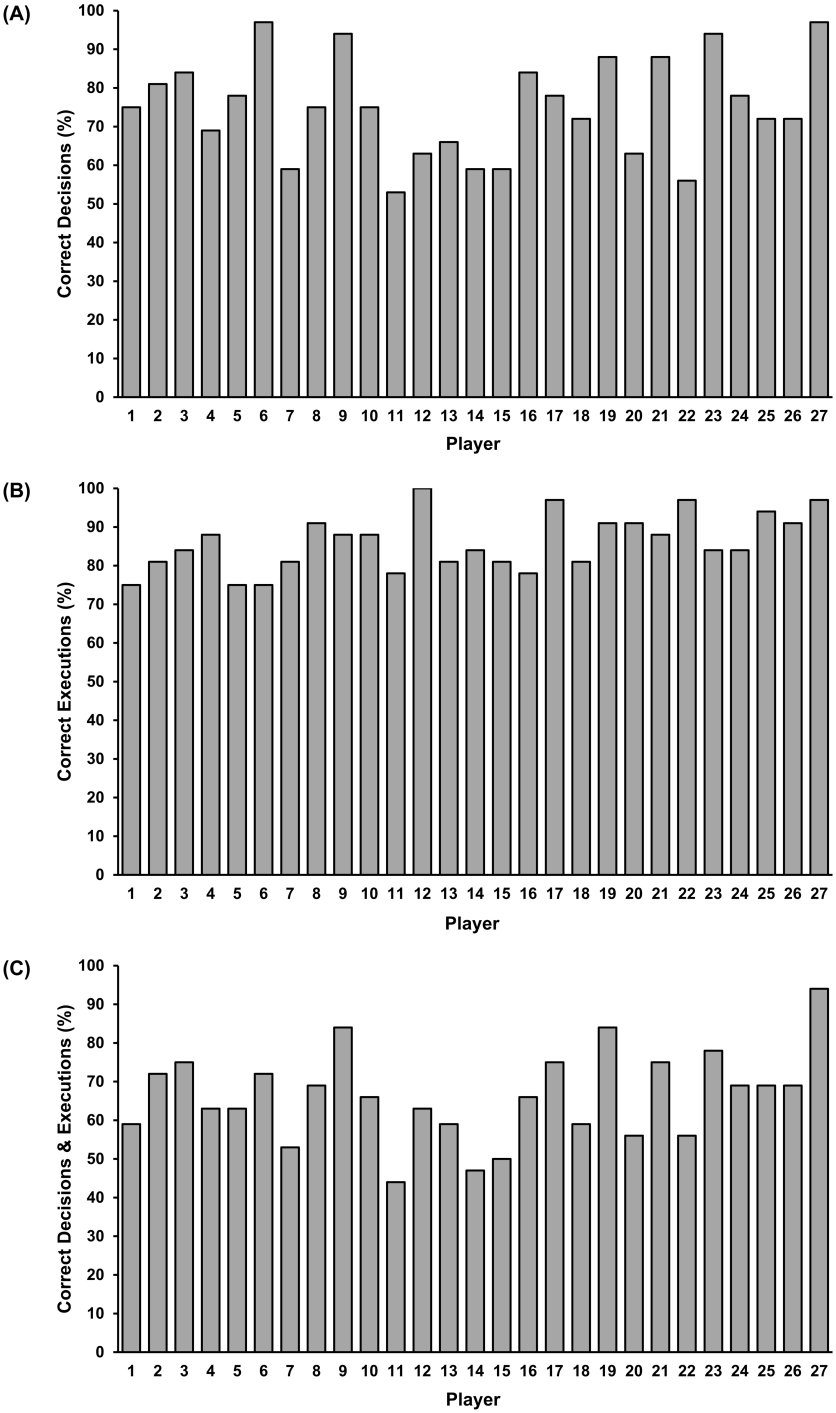
Players’ absolute percentage correct for decision-making **(A)**, execution **(B)**, and total **(C)**. Error bars are not plotted, as these are absolute values.

Figure 3A presents each player’s absolute percentage correct decision-making scores. For all players, out of all trials (*N* = 864), 75% (*n* = 649) were classified as good decisions and 25% (*n* = 215) were classified as poor decisions. GEE indicated that there were significant differences in decision-making between individual players, χ^2^ (26) = 340.25, *p* < 0.001. Post-hoc analyses indicated that players 6, 9, 23, and 27 were the cause of the significant differences between players’ decision-making accuracy (see Table 2).

**Table 2:** Absolute percentage differences [Wald 95% confidence intervals] for significant Post-hoc comparisons between players 6, 9, 23, and 27 and other players for decision-making accuracy.

Player	Player							
	7	11	12	13	14	15	20	22
6	37* [1, 74]	44** [10, 77]	-	-	38** [5, 70]	38* [1, 74]	34* [2, 66]	41* [4, 78]
9	-	41** [8, 74]	-	-	-	-	-	38* [1, 74]
23	34* [2, 66]	41** [8, 74]	-	-	-	-	-	38** [5, 70]
27	37* [1, 74]	44** [6, 81]	34* [2, 66]	31* [0, 62]	37* [1, 74]	37** [5, 70]	34* [2, 66]	41* [4, 78]

Values represent percentages. Positive values indicate superior performance of players 6, 9, 23, and 27 to comparison player(s), while negative values indicate inferior performance of players 6, 9, 23, and 27 to comparison player(s). Hyphen indicates no significant difference between players. *p < 0.05. **p < 0.01.

### 3.2 Execution accuracy

#### 3.2.1 Overall influence of age, experience, and playing level

**Figure 4:**
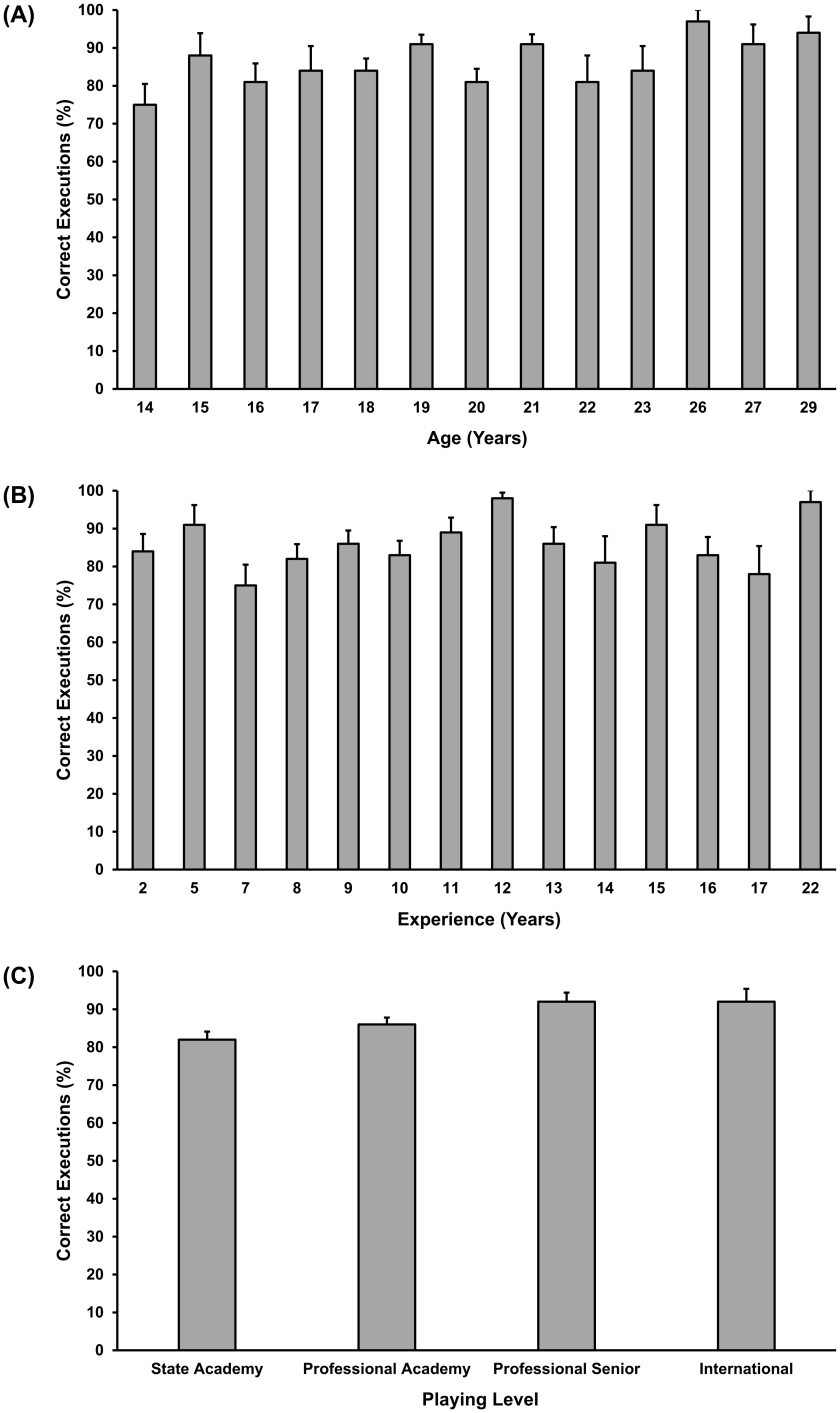
Mean percentage correct for execution relative to age **(A)**, experience **(B)**, and playing level **(C)**. Error bars indicate standard error.

Figure 4 presents mean execution scores relative to age, experience, and playing level. GEE indicated no significant effect of age [χ^2^ (1) = 0.16, *p* = 0.691], experience [χ^2^ (1) = 0.23, *p* = 0.630], or playing level [χ^2^ (3) = 2.55, *p* = 0.466] on execution accuracy.

#### 3.2.2 Inter-individual differences comparison

Figure 3B illustrates each player’s absolute percentage correct execution scores. For all players, out of all trials (*N* = 864), 86% (*n* = 743) were classified as good executions and 14% (*n* = 121) were classified as poor executions. GEE indicated that there were significant differences between individual players’ execution scores, χ^2^ (25) = 121.97, *p* < 0.001. However, post-hoc analyses did not detect significant differences between individual players’ execution scores.

### 3.3 Decision-making and execution accuracy

#### 3.3.1 Overall influence of age, experience, and playing level

**Figure 5:**
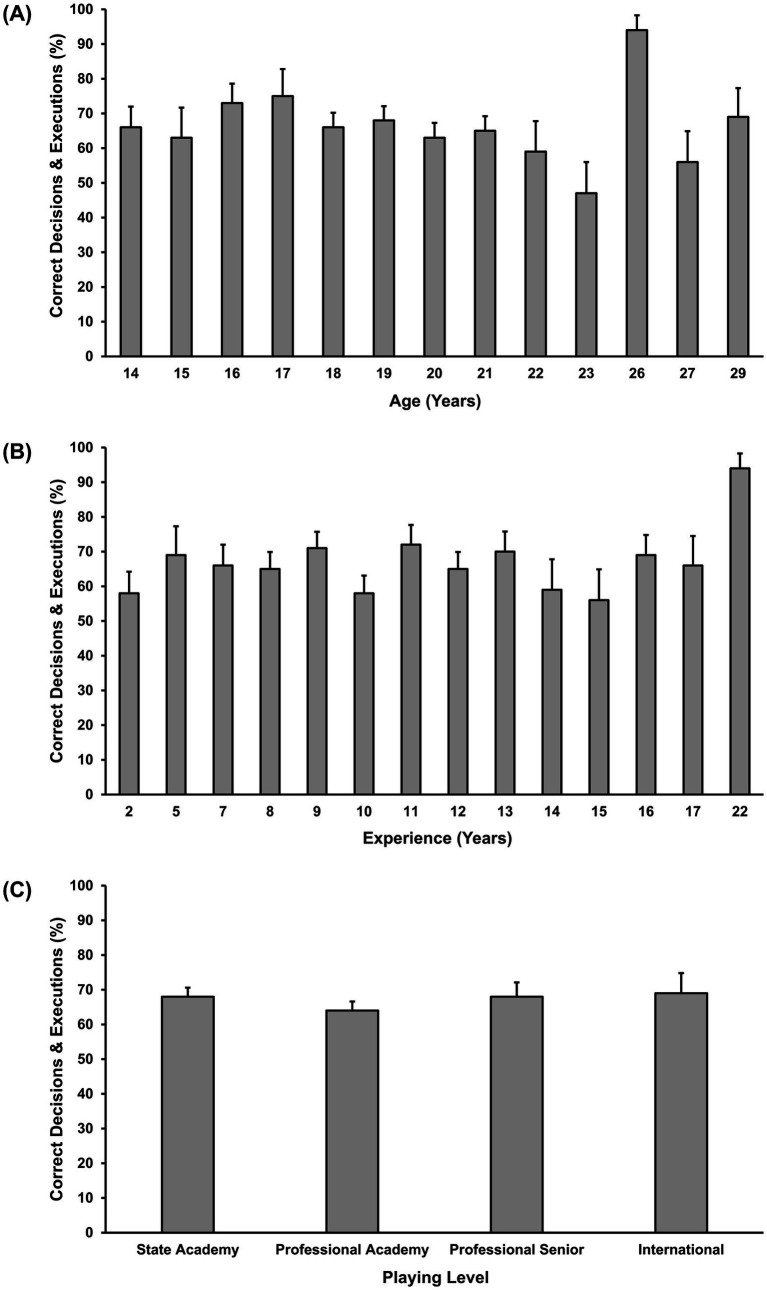
Mean percentage correct for total relative to age **(A)**, experience **(B)**, and playing level **(C)**. Error bars indicate standard error.

Figure 5 presents mean total scores relative to age, experience, and playing level. GEE indicated a significant effect of experience on total accuracy, χ^2^ (1) = 8.75, *p* = 0.003. An increase in experience by 1 year accounted for a 6% increase in the odds of making an accurate (good) decision and execution (OR = 1.06, 95% CI 2–11%). Further, GEE results revealed that age [χ^2^ (1) = 2.16, *p* = 0.141] and playing level [χ^2^ (3) = 4.28, *p* = 0.232] did not significantly influence total accuracy.

#### 3.3.2 Inter-individual differences comparison

Figure 3C presents each player’s absolute percentage correct total scores. For all players, out of all trials (*N* = 864), 66% (*n* = 572) were classified as trials with good decisions and execution and 34% (*n* = 292) were classified as trials with poor decisions and/or execution. GEE indicated that there were significant differences in total scores between individual players, χ^2^ (26) = 410.63, *p* < 0.001. Post-hoc analyses indicated that players 9, 11, 14, and 27 were the cause of the significant differences between players’ total scores. Players with significant differences (*p* < 0.05) to players 9, 11, 14, and 27 are presented in Table 3.

**Table 3:** Absolute percentage differences [Wald 95% confidence intervals] for significant post-hoc comparisons between players 9, 11, 14, and 27 and the other players for total accuracy.

Player	Player							
	1	7	11	12	14	15	19	20
9	-	-	41* [4, 78]	-	-	-	-	-
11	-	-	-	-	-	-	−41* [−78, −4]	-
14	-	-	-	-	-	-	−37* [−74, −1]	-
27	34* [2, 66]	41* [4, 78]	50** [9, 91]	31* [0, 62]	47** [9, 84]	44** [10, 77]	-	38* [1, 74]

Values represent percentages. Positive values indicate superior performance of players 9, 11, 14, and 27 to comparison player(s), while negative values indicate inferior performance of players 9, 11, 14, and 27 to comparison player(s). Hyphen indicates no significant differences between players. *p < 0.05. **p < 0.01.

## 4 Discussion

The purpose of this study was to investigate age, experience, and level played contributions to perceptual-cognitive-motor skill in a group of rugby players with high expertise using an in-situ test that was representative of competition. As all participants were in a rugby union academy or professional squad, this provided an opportune context for an individual differences fine-grained probe of perceptual-cognitive-motor skill, as skill level attained and experience with the task, were much closer than some previous studies in the literature. Findings suggest that greater experience is important but may not be the sole contributor to superior decision-making. Further, age and level played appear to have negligible influence on superior decision-making within a sample of athletes with high expertise. Importantly, the inter-rater reliability analysis indicated that the scoring of the field-test had a high level of reliability, which provides confidence that performance was scored consistently. This study therefore presents some important considerations for the conceptualization of expertise, as well as talent identification and skill development.

Investigation of the nature of individual differences across decision-making, execution, and both components combined, on the task was not the sole focus of this study. Nonetheless, it is important to consider that more pronounced inter-individual differences were found in the decision-making component of the task, than the execution component, which descriptively, seemed to drive combined task performance (see Figure 3). This indicates that even for athletes with high expertise, the perceptual-cognitive component, in this instant decision-making, appears to be a more crucial discriminator of performance, than motor execution (Piggott et al., 2019). Previous studies using a group-based design have indeed reported significant expert versus lesser-skilled differences in anticipation (e.g., Müller et al., 2010) and decision-making (e.g., Gorman et al., 2011; Piggott et al., 2019; Ashford et al., 2021). Our findings, however, contribute to the increasing number of studies that have reported perceptual-cognitive-motor differences within samples of athletes with high expertise (Morris-Binelli et al., 2021a; Lindsay and Spittle, 2024). Therefore, the inter-individual differences paradigms adopted here remains a worthwhile methodology to probe fine-grained differences in advanced and expert performers, which can be implemented across field-based or video and virtual reality simulation tasks.

It was predicted that an increase in player age would result in superior decision-making skill. The overall group-level analysis of the influence of age on decision-making did not support this hypothesis, as age was a significant negative predictor of decision-making. That is, an increase in age by 1 year resulted in an 11% decrease in the odds of making an accurate (good) decision. The inter-individual differences analysis provides further insight into the non-linear influence of age on decision-making skill. These findings indicate that players as young as 14 years old can possess decision-making capability at similar levels to a professional adult player who was aged 26 years (i.e., player 27). The findings of this study contrast with Abernethy (1988) and De Waelle et al. (2021) who reported advantages in perceptual-cognitive skill relative to adult performers only become apparent at under 17 years old or above, as well as Murr et al. (2021) who did not detect differences in decision-making between under 17 and under 19 players. Therefore, the individual differences approach implemented in this study had the sensitivity to identify junior players (i.e., player 6 and 9) with highly proficient decision-making skill.

It was also predicted that those players who had attained a higher level of play (expertise) in the sport of rugby union would consistently have superior decision-making skill. The findings of this study, however, did not support this hypothesis. The overall group-level analysis reported that playing level was not a significant predictor of decision-making. This finding contrasts the broader literature on anticipation and decision-making, which reports that groups of performers with higher levels of expertise outperform groups of performers with lower expertise in sports such as basketball (e.g., Aglioti et al., 2008; Gorman et al., 2013), soccer (e.g., Murr et al., 2021), and Australian rules football (e.g., Piggott et al., 2019; Panchuk and Maloney, 2022). Like for age, the inter-individual differences analysis provides further insight into the non-linear influence of playing level on decision-making skill. For example, players 6 and 9 who are considered playmakers, but had not played professional senior rugby union, achieved decision-making performance that was comparable to player 27, who was also a playmaker and competed at professional senior level (see Table 1; Figure 3). Therefore, an inter-individual differences approach provides a more nuanced understanding of the complex nature of decision-making skill as it has the sensitivity to identify players with superior decision-making skill regardless of their playing level (expertise).

Finally, this study predicted that those players who had higher levels of experience in rugby union would have superior decision-making skill. The overall group-level analysis supported this hypothesis as experience was a significant predictor of decision-making skill, with an increase in experience of 1 year increasing the odds of making an accurate (good) decision by 8%. This finding aligns with the literature on anticipation and decision-making in sport which reports performers with greater experience outperform performers with less experience on domain-specific tasks (Farrow and Abernethy, 2015; Raab and Helsen, 2015; Williams and Jackson, 2019). Interestingly, although experience significantly predicted decision-making, based upon the odds ratio, its influence could be viewed as relatively low. The inter-individual differences analysis again provides unique insight into the degree of influence that experience has on decision-making. In line with the overall group-level analysis, the inter-individual differences analysis provided some support for the experience hypothesis because player 27 who had the most experience compared to the rest of the sample (see Table 1), scored the highest in terms of decision-making (see Figure 3). However, players 6 and 9 performed similarly to player 27 despite considerably less experience in rugby union (see Table 1; Figure 3). Further, player 6 significantly outperformed player 14, who was also considered a playmaker, despite 3 years less experience in rugby union. Finally, individual participant’s decision-making accuracy scores ranged from 53 to 97% (see Figure 3). These findings align with previous research that has used an inter-individual differences approach to investigate perceptual-cognitive-motor skill (e.g., Land and McLeod, 2000; Morris-Binelli et al., 2021a). That is, the individualized nature of anticipation skill in striking sports that does not appear to be solely dependent upon increased experience extends to decision-making skill in invasion sports.

There are important theoretical and practical implications from the findings of this study. In relation to theory, it has been suggested based upon the deliberate practice framework that attainment of expert performance is due to monotonic and linear increases in domain-specific exposure (Ericsson et al., 1993; Ericsson, 2020). While the findings of this study indicate that greater experience within the domain sport does to a degree contribute to superior decision-making skill, relatively less experience within the domain did not limit attainment of high decision-making capability. In a related manner, there are studies in the development of sport expertise literature that have reported both prolonged engagement within (Roca et al., 2012; Hendry et al., 2018), and later specialization in (Baker et al., 2003), a sport can contribute to superior decision-making skill. Moreover, recent studies have reported that superior perceptual-cognitive skill and coach rating of skill creativity is not necessarily related to linear progressions from lesser-skilled to expert levels (Hendry et al., 2018; Morris-Binelli et al., 2021a). Accordingly, rather than conceptualize perceptual-cognitive-motor expertise as a by-product of greater exposure within a domain, it would seem more appropriate to consider expertise as the capability to search for, and pick-up, informative perceptual information, and be able to transfer that capability from one rapidly changing context to the next in order to guide action (Müller and Rosalie, 2019; Güllich et al., 2022). Such a conceptualization of skill acquisition and expertise as non-linear (see, e.g., Chow et al., 2016; Pacheco and Newell, 2018) and incorporating transfer of skill from one instant to another has the potential to provide a more balanced perspective to perceptual-cognitive-motor skill expertise, which will also avoid a focus upon prolonged exposure that can cause fatigue, injury, or burnout (Müller et al., 2023).

In relation to practical application, representative field-based tests like the one used in this study can be easily co-designed between coaches and skill learning specialists. Incorporating nested inter-individual differences comparisons provides a powerful way to explore strengths and deficiencies in the perceptual-cognitive-motor capability of athletes. Thereafter, a variety of training approaches can be used to target improvement in deficiencies, such as through video-based and virtual reality training (Fortes et al., 2021) or motor practice of what is to be perceived (Brenton et al., 2019). Further, a more nuanced understanding of athletes’ perceptual-cognitive-motor skill within a team or developmental pathway, may better inform talent identification and development as well as coaches’ tactical decisions to increase the chance of team success. Professional sports such as rugby union in this paper are investing more time and resources into this approach, which has been reported by coaches as an important part of competition preparation (Morris-Binelli et al., 2021b).

## 5 Conclusion, limitations, and future research

This study investigated the influence of age, experience, and level played on advanced and expert rugby union players’ perceptual-cognitive-motor skill within an in-situ test using an inter-individual differences paradigm. Decision-making, rather than motor execution, appeared to be the main discriminator of performance. The capability to make superior decisions is unlikely to be solely dependent upon increased experience, while age and level played appear to have little influence on decision-making skill within a sample of athletes with higher expertise. This indicates that caution should be exercised in the conceptualization of expertise as monotonic and linear in nature, as well as highlights the importance of individual differences in explaining performance capabilities of athletes in talent development pathways and high-performance programs (Morris-Binelli et al., 2021a). A potential limitation of this study is that it was restricted to a specific play context of a rugby union match. Therefore, within athletes of high expertise, age, experience, and level played may influence decision-making differently in other contexts of a rugby union match or in other invasion sports, such as soccer. Nonetheless, coaches agreed that off the ruck scenarios are a crucial and frequent decision-making part of rugby union match play. In-situ research is also challenging to complete due to the time requirement of several participants, so multiple game scenarios could not be implemented. In future research, perceptual-cognitive-motor skill in other representative rugby union game scenarios and other invasion sports could be investigated to develop a more complete understanding of age, experience, and skill level to performance. For example, ‘good’ and ‘poor’ decision-makers across the skill continuum could be identified using an inter-individual differences approach and then their practice and competition histories analyzed to further understand key determinants of decision-making skill. In addition, future studies could investigate the capability of athletes to integrate contextual (e.g., game score and time) and kinematic (e.g., teammate and opponent movement pattern) information for decision-making across a high-performance pathway at the individual level. Based upon the findings in this study, it is likely important that talent scouts and coaches consider that exceptional performance may appear at relatively younger or older ages with varying degrees of experience within a domain. Such a broad focus may allow better capture of a larger talent pool through tracking of earlier or later developing players for skill training.

## Data Availability

The datasets presented in this article are not readily available because athlete data cannot be shared. Requests to access the datasets should be directed to: khaya.morris-binelli@nd.edu.au.
