# Supplementary material for: Individual differences provide a nuanced understanding of the contributions of age, experience, and level played to superior perceptual-cognitive-motor skill
Source: Front Psychol. 2025 Apr 22;16:1470789. doi: 10.3389/fpsyg.2025.1470789 (PMC12053291; doi:10.3389/fpsyg.2025.1470789)
Supplement: Supplementary file 1 [file Data_Sheet_1.pdf]

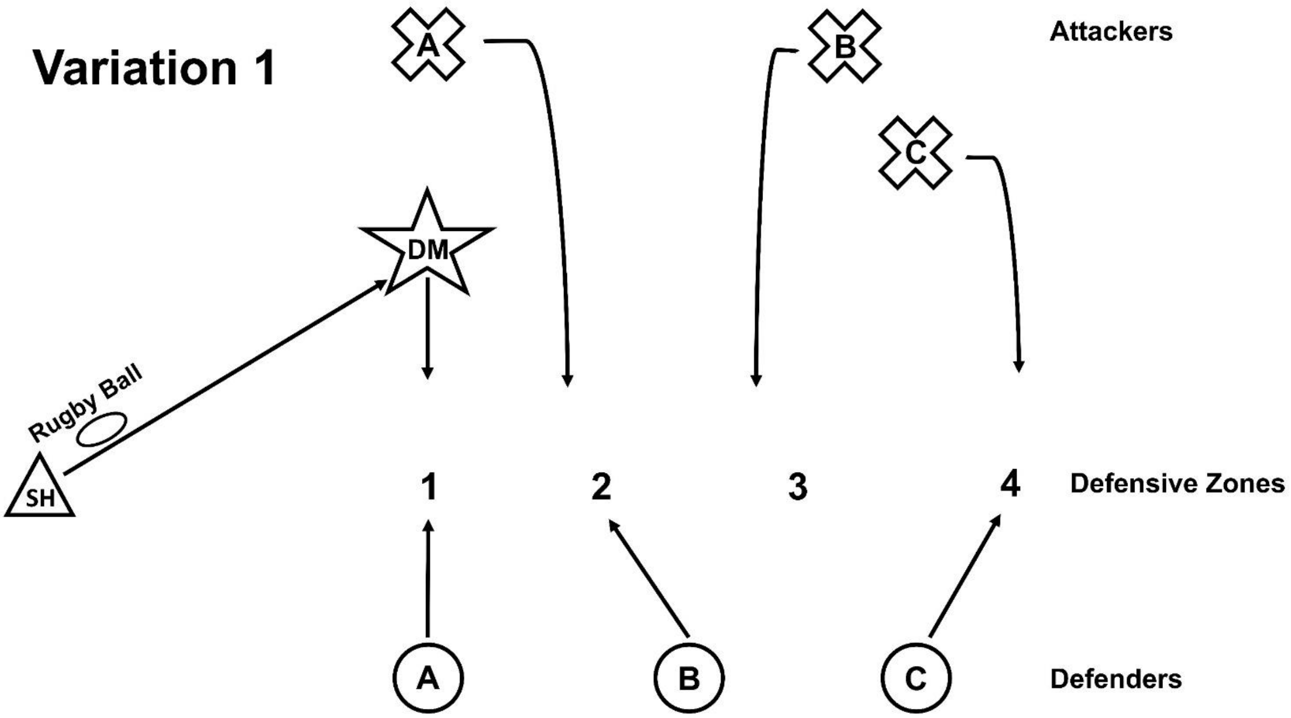

Supplementary Figure 1. Schematic of play variation one in the rugby decision-making field test (passing left).

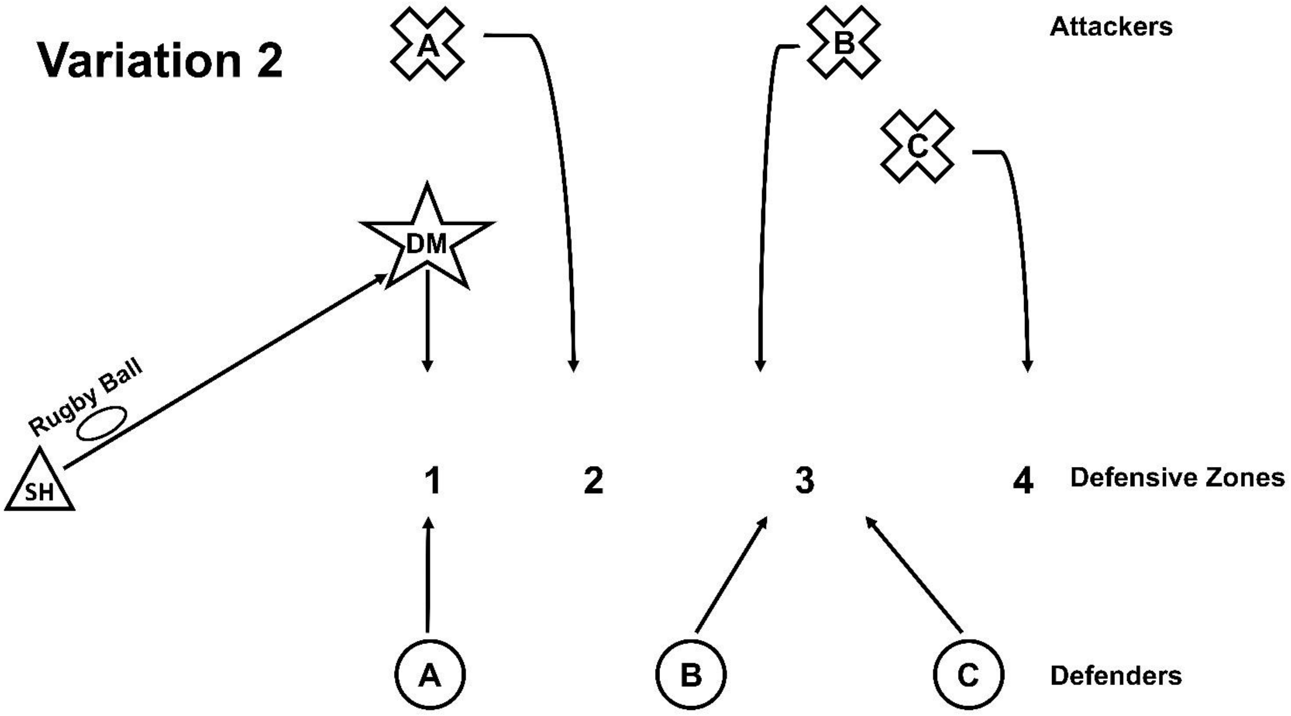

Supplementary Figure 2. Schematic of play variation two in the rugby decision-making field test (passing left).

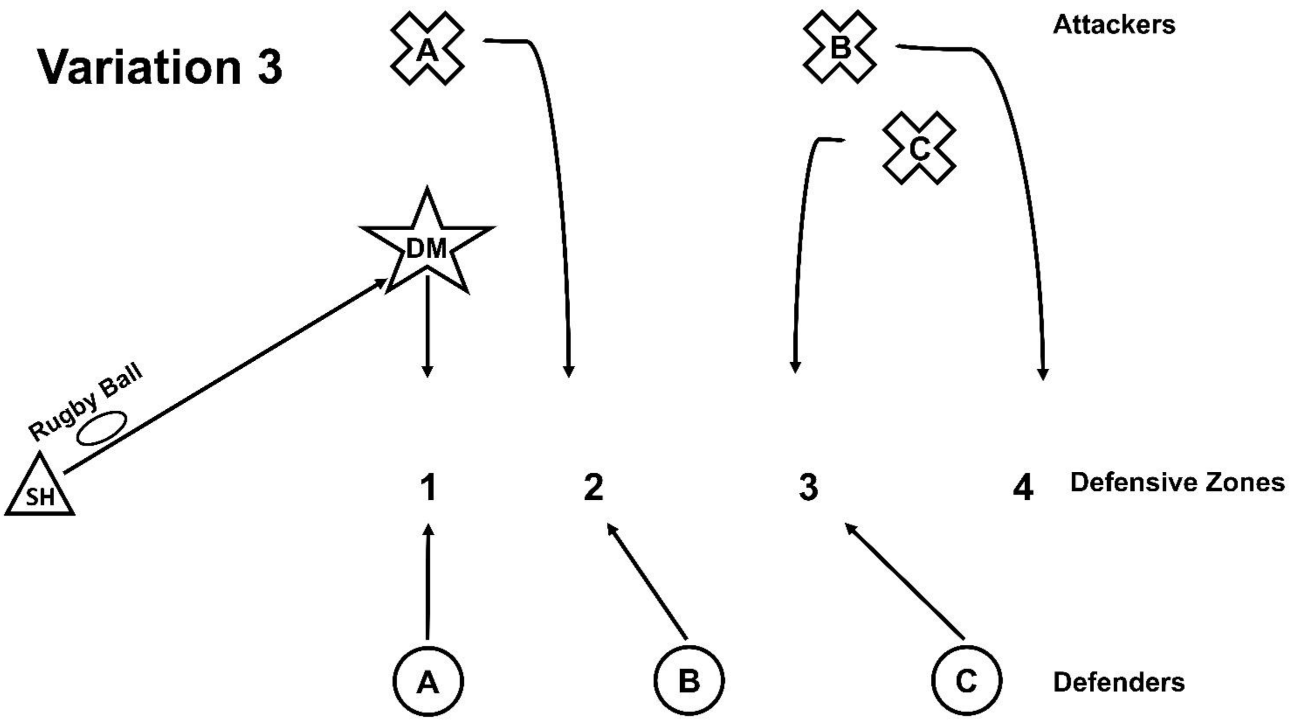

Supplementary Figure 3. Schematic of play variation three in the rugby decision-making field test (passing left).

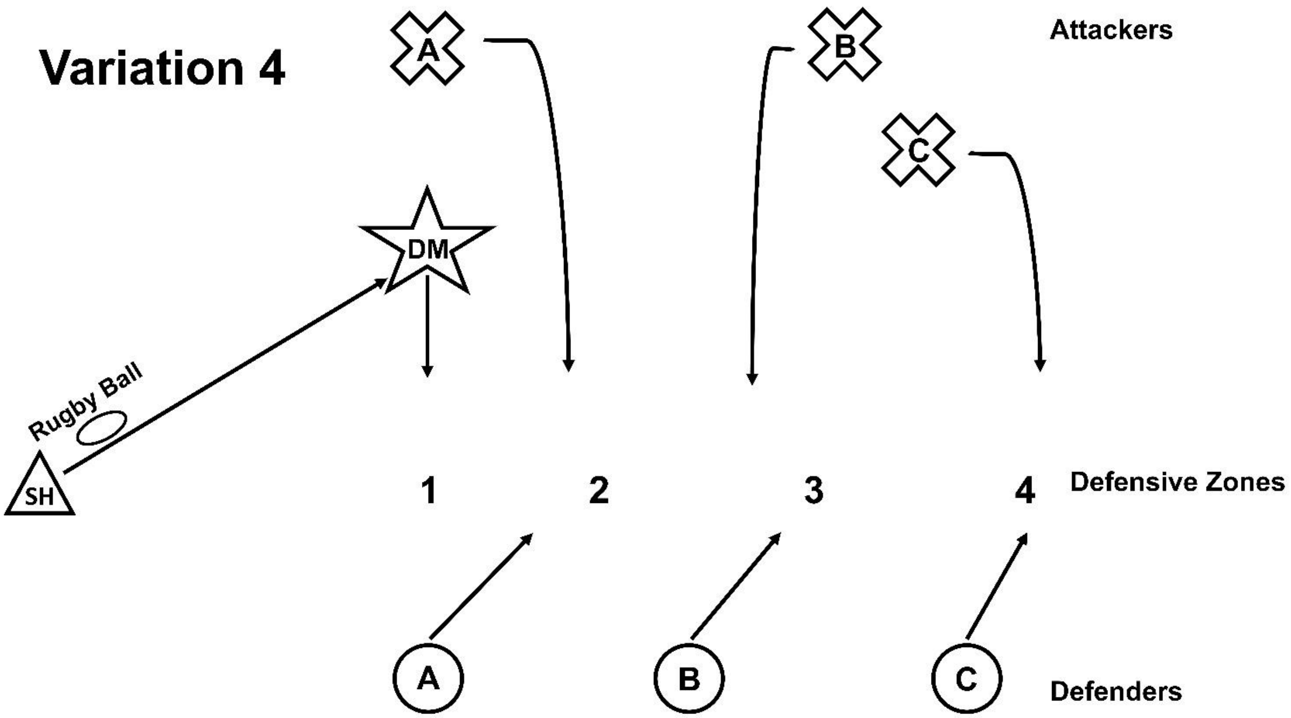

Supplementary Figure 4. Schematic of play variation four in the rugby decision-making field test (passing left).

Supplementary Table 1. Scoring for all defensive variations for decision-making and execution.

Defense Variations	Respective Decision Scoring (1 = Good, 0 = Poor)	Pass Execution Scoring for All Variations (1 = Good, 0 = Poor)	Run Execution Scoring for All Variations (1 = Good, 0 = Poor)
**1**	**(1)** Pass to attacker B or drive forward<br>**(0)** Pass to attacker C or pass to attacker A	**(1)** Ball reaches target on full & target does not have to break stride or ball reaches target on full, however, target has to break stride/change direction/over-reach for ball<br>**(0)** Ball reaches target, but not on full; ball thrown with too much force or ball does not reach target at all	**(1)** No body contact with defender, hard explosive run, definitive drive or explosive but touched by defender<br>**(0)** Low intensity/uncertain in decision or no definitive movement/stops moving
**2**	**(1)** Pass to attacker A or pass to attacker C<br>**(0)** Drive forward or pass to attacker B		
**3**	**(1)** Pass to attacker B or pass to attacker C<br>**(0)** Drive forward or pass to attacker A		
**4**	**(1)** Drive forward or pass to attacker B<br>**(0)** Pass to attacker A or pass to attacker C		
